# Utility of Fluorescent Microscopy for Rapid Intraoperative Diagnosis of Rhinocerebral Mucormycosis: Experience from a Tertiary Care Center in Central India

**DOI:** 10.7759/cureus.81537

**Published:** 2025-03-31

**Authors:** Nisha Meshram, Sachin Chaudhari, Milind Bhatkule, Rasika Gadkari

**Affiliations:** 1 Department of Pathology, All India Institute of Medical Sciences, Nagpur, Nagpur, IND

**Keywords:** calcofluor white, covid-19, fluorescent stain, frozen section, koh, rhinocerebral mucormycosis

## Abstract

Rhino-orbital-cerebral mucormycosis is an uncommon life-threatening infection caused by the angioinvasive fungus *Mucorales *and is associated with high morbidity and mortality. In India, the pandemic of COVID-19 was associated with another deadly disease, rhinocerebral mucormycosis, which further complicated the course of the disease, necessitating an accurate and rapid diagnosis. Conventional methods of diagnosis, like fungal culture, histopathology, Gomori methenamine silver (GMS), and periodic acid-Schiff (PAS) stain, are not feasible for intraoperative diagnosis. We studied the use of fluorescent brightener calcofluor white (CFW) for rapid intraoperative diagnosis of mucormycosis and compared it with intraoperative crush smear cytology and frozen section. A total of 37 intraoperative samples were studied, of which 11 were positive for the fungus. Calcofluor white detected fungus in seven samples, while frozen section detected fungus in eight samples. Calcofluor white stain showed less sensitivity than the frozen section but had high specificity. In the presence of marked necrosis, suspicious fragments on frozen sections could be quickly confirmed by fluorescent stain. Thus, CFW direct microscopy is a useful adjunct for the rapid diagnosis of mucormycosis.

## Introduction

From December 2019 to early 2023, the world experienced a deadly pandemic of COVID-19 with associated mortality and morbidity. India experienced a deadly second wave from around March 2021 to June 2021, which presented with more severe disease, resulting in increased use of invasive support systems and immunomodulation [1-4]. But in all this mayhem, no one could guess a new danger lurking around the corner, the epidemic of rhinocerebral mucormycosis [4]. Globally, the highest number of cases were reported from India, amounting to more than 4,000 people with COVID-19-associated mucormycosis (CAM) [5-7].

Mucormycosis most commonly involves the rhinocerebral, pulmonary, gastrointestinal, and subcutaneous tissues [8]. This disease is angio-invasive and causes rapid progression with tissue destruction, facial cellulitis, gangrenous mucosal changes in the nose and paranasal sinuses, with orbital and intracranial extension and death [6]. The success of therapy depends on early detection, systemic antifungal therapy with liposomal amphotericin B, along with aggressive surgical debridement of affected tissue causing cosmetic disfigurement [9]. Among the survivors, vision loss followed by facial deformity is a frequently encountered consequence of mucormycosis [7]. Thus, the earliest diagnosis and immediate treatment are of utmost importance. Song et al. have suggested an algorithm for the early diagnosis and management of common invasive fungal infections (*Aspergillus*, candidiasis, cryptococcosis, and mucormycosis) [10].

Various methods for laboratory diagnosis of mucor include direct microscopy, cytology, histopathological examination, culture, and molecular methods like polymerase chain reaction (PCR)-based kits, fluorescent in-situ hybridization (FISH), or immunohistochemistry (IHC) on formalin-fixed paraffin-embedded tissue blocks [11-13]. Frozen section as a diagnostic method can be used in patients with rhino-cerebral mucormycosis in two scenarios: (1) to perform a rapid diagnosis allowing immediate administration of antifungal therapy, and (2) to guide complete eradication of the infected tissue during the surgical debridement [14]. Routine stains for fungus, like Gomori methenamine silver (GMS) and periodic acid-Schiff (PAS), are time-consuming and technique-sensitive and hence cannot be used for intraoperative decision-making.

Calcofluor white (CFW), also called a fluorescent brightener, optical brightener, or “whitening agent,” is routinely used in the paper, textile, and related industries as an agent to whiten the papers and fabrics [15]. Calcofluor white binds specifically to the beta (β) 1‑3, β 1‑4 glycoside chain of the chitin in the fungal cell wall, which almost immediately fluoresces under ultraviolet (UV) light with an intense apple green or bluish color against the white background, depending on the filter used. The addition of potassium hydroxide (KOH) in the CFW stain increases the visibility of the fungal elements in clinical samples [16].

The use of CFW stain for the detection of fungus was first described by Hageage and Harrington [17]. Since then, it has been used by various researchers for the demonstration of fungi in different clinical samples [18-21]. McDermott et al. used calcofluor-stained tissue as a rapid technique for intraoperative diagnosis of fungus and for assessment of margin clearance during frozen sections [12, 18]. Thus, the present study was undertaken to compare the efficacy of intraoperative CFW stain direct microscopy with intraoperative crush smear cytology and frozen sections for rapid diagnosis of rhinocerebral mucormycosis and other fungal infections in COVID-19-positive and post-COVID patients.

Part of this article was previously presented as a meeting abstract at the 50^th^ Annual Conference of the Indian Academy of Cytologists, held virtually from 19 to 21 November 2021.

## Materials and methods

Study design

This was a prospective, diagnostic test evaluation study performed over a period of three months, from May 2021 to July 2021, at the Department of Pathology of All India Institute of Medical Sciences, a tertiary care institute in Nagpur, India.

Study population

Inclusion Criteria

Intraoperative samples from suspected cases of COVID-19-associated rhinocerebral mucormycosis for frozen section diagnosis were included.

Exclusion Criteria

All other samples, like smears from nasal secretions, nasal swabs, sputum, or tracheal secretions, etc., were not included in the study.

Sample Size

A total of 37 samples from suspected cases of mucormycosis were included in the study; 22 were from male patients and 15 were from female patients.

Methodology

Endoscopic resection specimens or biopsies received for intraoperative diagnosis of fungal infection were divided into three parts. i) A small portion of the sample, preferably from the necrotic or blackish area, was used for the preparation of crush smears on a clean, grease-free slide, which was used for the preparation of calcofluor white wet mount. ii) Another small portion of the sample was used to prepare a crush smear, which was stained with hematoxylin and eosin stain (H&E) by the rapid method. These smears were called smears for intraoperative crush smear cytology. iii) The rest of the tissue was submitted for frozen section evaluation and stained with H&E by the rapid method.

Once the diagnosis on the frozen section was made & conveyed to the surgeon, the frozen tissue was submitted for routine histological processing for making formalin-fixed paraffin-embedded (FFPE) tissue blocks. Sections were cut and stained with PAS and H&E stain, and the sections were studied under a microscope for the presence of fungal elements. Histopathology diagnosis was considered the gold standard.

Calcofluor white stain wet mount preparation (direct microscopy)

One drop of ready-to-use CFW (Sigma Aldrich, St. Louis, MO), comprising 1 g/L CFW M2R, was added to the crush smear slide, followed by one drop of 10% KOH, and covered with a cover slip. The staining procedure was carried out in a darkroom setting. The CFW-KOH wet mount preparation was then examined under a fluorescence microscope (Leica DM750, Leica Microsystems, Wetzlar, Germany) using a blue light excitation filter, which has a 300-400 nm emission wavelength with excitation at around 355nm [15].

The procedure for the rapid staining method of H&E is outlined in Table 1.

**Table 1:** Procedure for the rapid staining method of hematoxylin and eosin stain

Step no.	Procedure	Time
1.	Immediately fix frozen sections in 95% ethyl alcohol	15 seconds
2.	Transfer to formalin 10%, phosphate buffered	10 dips
3.	Rinse well in distilled water	10 dips
4.	Stain with hematoxylin stain, Harris modified	30 seconds
5.	Wash well in two changes of distilled water	10 dips each
6.	Place in 95% ethyl alcohol	10 dips
7.	Counterstain in eosin Y working solution.	15 sec
8.	Dehydrate in two changes of 95% ethyl alcohol	10 dips each
9	Dehydrate in two changes of 100% ethyl alcohol	10 dips each
10.	Clear in two changes of xylene	10 dips each
11.	Coverslip with compatible mounting medium	

Interpretation

Calcofluor White Stain Wet Mount Preparation (Direct Microscopy)

**Figure 1:**
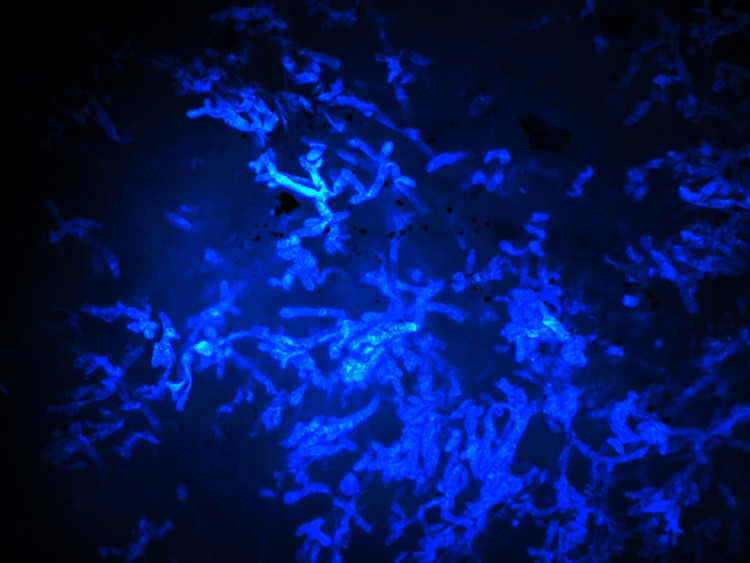
Calcofluor white stain under fluorescent light showing irregular, non-septate hyphae (original magnification 40x).

The hyphae of *Mucor *stained blue against the black background. The fungal hyphae were sharply delineated and could be identified even on the scanner view. Brighter staining was observed when the calcofluor stain was allowed to set for a longer duration (Figure 1).

Intraoperative Crush Smear Cytology

**Figure 2:**
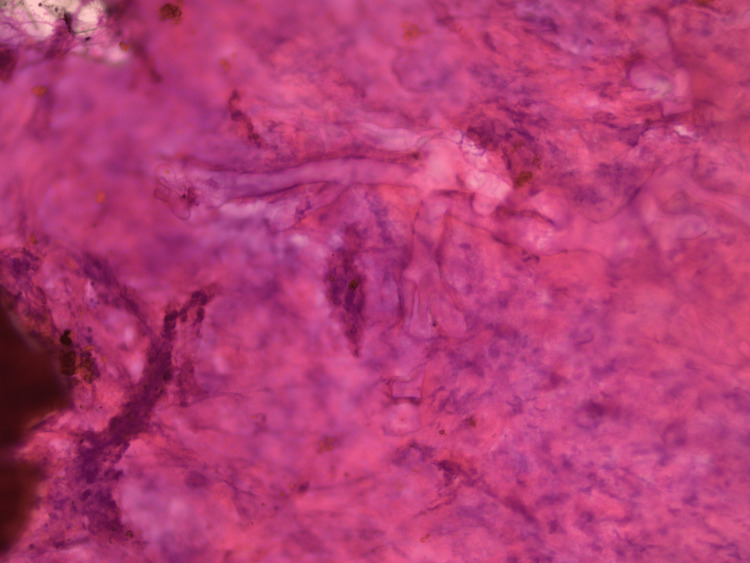
Intraoperative crush smear cytology showing mucor hyphae, which are broad, ribbon-like and non-septate (H&E; original magnification 400X).

The cytology smears showed broad, ribbon-like aseptate fungal hyphae with right-angled branching at irregular intervals. Many twisted and wrinkled forms were also seen (Figure 2).

Frozen Sections

**Figure 3:**
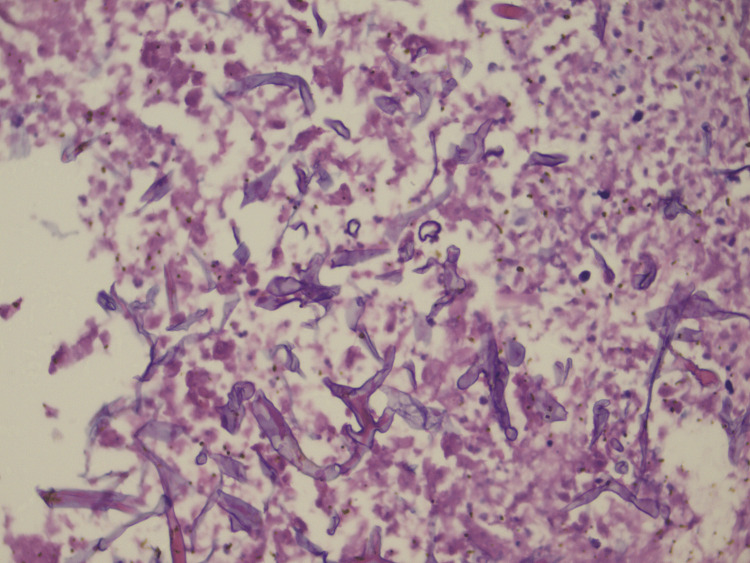
Endoscopic biopsy tissue on frozen section showing invasion by mucor hyphae. Many twisted and bizarre forms are also seen in the background of necrotic material (H&E; original magnification 400X).

On frozen tissue, *Mucor *was seen as broad, aseptate, ribbon-like, thin-walled hyphae with right-angled, irregular branching. Twisted, collapsed, wrinkled, and single hyphal forms were also seen (Figure 3).

Histopathology

The hyphae stained a faint blue with PAS stain. 

## Results

A total of 37 samples from cases suspected of mucormycosis were included in the study; 22 were from male patients and 15 from female patients. Of the 37 samples, 11 (30%) were positive for fungus while 26 (70%) were negative. Of the 11 samples positive for fungus, 10 (90%) showed the presence of mucormycosis, while one (10%) was positive for *Aspergillus*.

Of 11 positive samples, seven (63.6%) were positive with direct microscopy by CFW stain, while only four (36.3%) samples detected fungus on intraoperative crush smear cytology. Eight (72.7%) samples showed fungus on frozen tissue.

One sample was positive for fungus on the CFW stain wet mount, but did not show any fungus on frozen tissue. However, the routine paraffin sections from the same sample showed the presence of fungus. Two samples were negative by both CFW stain wet mount and crush smear cytology but showed fungus on frozen tissue and were reported as positive.

Three samples were negative for fungus on intraoperative tissue by all three methods, i.e., CFW stain wet mount, crush smear cytology, and frozen section, but showed fungus on routine histopathology sections.

Of the 26 negative samples on frozen tissue, separate (more) tissue was received in 25 cases, of which eight showed the presence of fungus. Seven samples showed *Mucor*, while one showed a mixed fungal infection of *Mucor *and *Aspergillus*. This disparity could be due to sampling error during freezing.

The sensitivity of the CFW stain wet mount was 63.6%, while specificity was 100% for the detection of fungus (Table 2). The positive predictive value (PPV) was 100%, while the negative predictive value (NPV) was 86.7%. The sensitivity of the frozen section was 72.7%, while the specificity was 100%. The PPV was 100% and the NPV was 89.66%. However, the sensitivity of intraoperative crush smear cytology was only 26.67%, but the specificity was 100%.

**Table 2:** Comparison of calcofluor white direct microscopy, intraoperative crush smear cytology and frozen section as test methods as compared to the gold standard (histopathology; n=11)

Test	Positive samples	Sensitivity	Specificity
Calcofluor white stain	7	63.6%	100%
Intraoperative crush smear cytology	4	26.6%	100%
Frozen section	8	72.7%	96.3%

## Discussion

Rhinocerebral mucormycosis is reported to be exclusively seen in immunocompromised hosts, including uncontrolled diabetes mellitus, hematologic cancers, and solid organ or hematopoietic stem cell transplants [9]. A study on 929 patients, done over 60 years, found poorly controlled diabetes mellitus as the most common risk factor (36%), followed by hematologic cancers (17%) and hematopoietic stem cell or solid organ transplant (12%) [22].

The development of CAM, uncontrolled diabetes, and systemic corticosteroid (over)treatment were the major risk factors in India and other countries [5]. Nonjudicious use of antifungal agents, especially voriconazole as a prophylactic agent, further enhanced the possibility of mucormycosis. COVID‑19 infection itself is a predisposing risk factor as it causes lymphopenia and endothelitis, favoring the fungal invasion [3, 13]. While disease manifestation and incidence vary considerably between different geographical regions [7], rhino-orbital-cerebral and pulmonary diseases are the most frequent clinical presentations of CAM in patients with COVID-19.

To the best of our knowledge, this is probably the first study using CFW stain wet mount on intraoperative crush smears for the detection of rhinocerebral mucormycosis. Other authors have studied its use for the detection of fungal agents in different tissues and samples.

It was Darken, who in 1961 reported the uptake of CFW by growing yeast and higher fungi [23]. Hageage and Harrington outlined the application of CFW to demonstrate hyphae and yeasts in clinical specimens in 1984 [17]. Monheit et al. used it on lung and soft tissues for the intraoperative diagnosis of fungal infection. In 1986, Monheit et al. recommended the incorporation of CFW with routine Papanicolaou stain on cytology for the identification of the fungus. They reported that it increases both the sensitivity and specificity of the stain [24]. Also, it doesn’t interfere with the diagnostic value of the Papanicolaou stain, and CFW does not distort or destroy cytopathologic features as done by GMS and PAS stains [19]. As CFW fluoresces under UV light, it is easily visible in tissue samples. Also, it can be used on all types of samples, such as fresh, fixed, and frozen tissue and paraffin-embedded tissue blocks [15]. Evans blue dye can be used as a counterstain with CFW, as it reduces the background fluorescence.

The hyphae of *Mucor *are variable in size, averaging 15-20 μm in diameter, and are aseptate. However, the creases and folds in the distorted hyphae resemble septa. When the tissue is handled or cut, the fluid contents of the hyphae escape, and they appear as hollow tubes or as twisted ribbons because of the folding of the empty tubes. The hyphae of *Aspergillus *are thin, slender, and have acute-angled branching [20].

**Figure 4:**
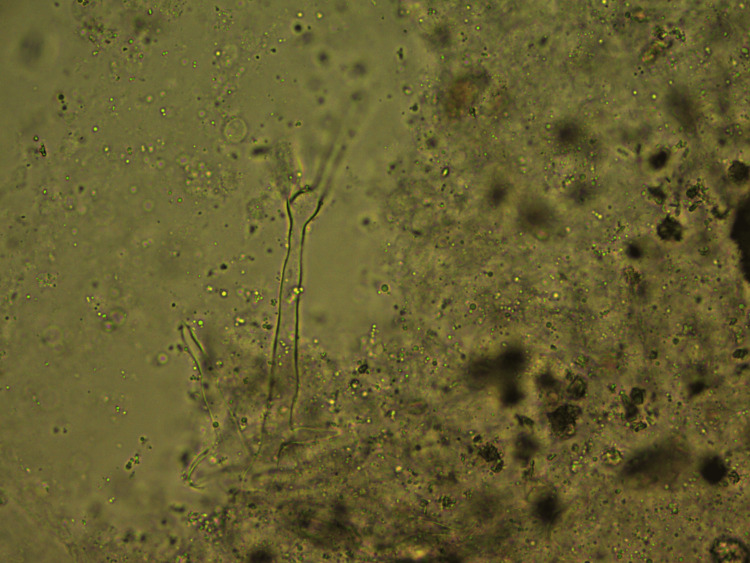
Calcofluor white stain wet mount under light microscopy showing irregular non-septate hyphae in the background of debris (original magnification 400X).

**Figure 5:**
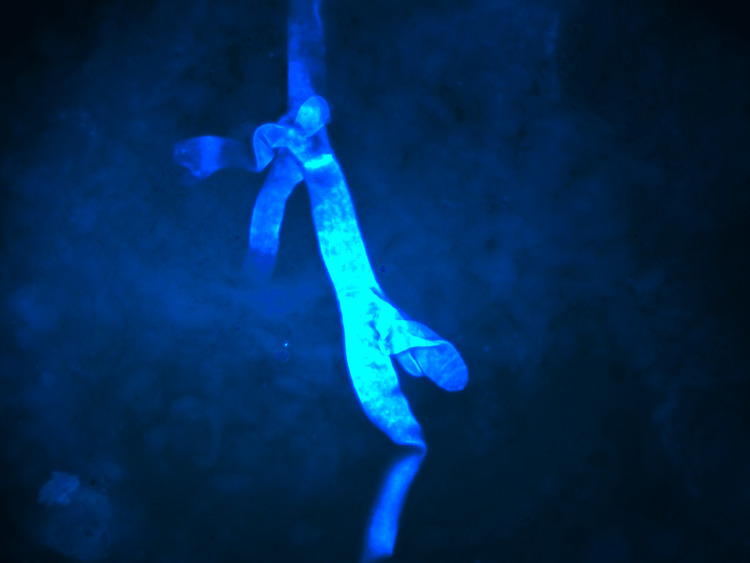
Calcofluor white stain wet mount under light microscopy showing irregular non-septate hyphae in the background of debris (original magnification 400X).

In our study, out of 37 samples, 11 (30%) were positive for fungus while 26 (70%) were negative. Of the 11 cases positive for fungus, 10 (90%) showed the presence of mucormycosis, while one case (10%) was positive for *Aspergillus*. Of 11 positive samples, seven (63.6%) were positive with direct microscopy by CFW stain wet mount. When a CFW wet mount is observed under a brightfield microscope, the presence of cellular debris and necrotic material may obscure the fungus and cause a delay in identification, but under the fluorescent microscope, the fungus fluoresces brightly and is sharply delineated (Figures 4, 5).

Intraoperative crush smear cytology detected fungus in only four (36.3%) samples. The presence of necrotic cellular material, causing difficulty in the visualization of hyphae, could be one of the reasons for the lower detection rate. Also, due to time constraints, the smear was not observed as thoroughly as needed.

Eight (72.7%) samples showed fungus on frozen tissue. One sample was positive with the CFW stain wet mount, but did not show any fungus on frozen tissue. However, the routine paraffin sections from the same sample showed the presence of hyphae. This could be due to the fact that hyphae were hidden in the deeper part of the tissue, which was not exposed during sections taken for frozen sections, while for CFW stain, we selected preferably the necrotic, blackish tissue. Two samples were negative by both CFW stain wet mount and intraoperative crush smear cytology but showed fungus on frozen tissue and were reported as positive. This could be due to selective sampling for the wet mount. On frozen sections, mucor stains faint blue, and many twisted or bizarre forms are seen. Oedematous nasal mucosa combined with water artifacts in frozen can be confused with fungus when mixed with inflammatory cells and debris. Hence, an experienced pathologist is needed for the interpretation of frozen sections.

In the present study, the sensitivity of the CFW stain wet mount was 63.6%, while the specificity was 100% for the detection of fungus. The PPV was 100%, while the NPV was 86.7%. Four cases that were missed could be due to sampling error, as only a part of the tissue received was observed. The sensitivity of the frozen section was 72.7%, while the specificity was 100%. The PPV was 100% and the NPV was 89.66%. However, a prospective study of mucormycosis in north India by Bala et al. mentions that the detection rate of mucormycosis by CFW stain (84%) was better than histopathology (58%) and culture (61%) [25].

Hofman et al. demonstrated that the frozen section is a specific and sensitive method for early diagnosis of rhino-cerebral mucormycosis and improves the prognosis of this disease. Cytological imprints performed on surgical biopsies were less effective than the frozen section for the immediate diagnosis of mucormycosis because of the presence of hemorrhages and necrotic tissue. Inexperienced pathologists may miss the diagnosis of rhinocerebral mucormycosis during the frozen section when the *Mucorales *show some bizarre forms and are twisted [14].

Sharma et al. studied CFW stain for the diagnosis of corneal keratitis and found CFW stain to be superior over KOH with a positivity rate of 100% and 87.5%, respectively [19]. Lackner et al. also recommended the use of optical brighteners, blankophor, and CFW in clinical specimens for a rapid diagnosis of mucormycosis [26]. Shakthi et al. in their comparative study of KOH, CFW stain, and fungal culture in cases of onychomycosis found that the sensitivity and specificity of KOH wet mount was 83.05%, whereas CFW microscopy showed a sensitivity of 92.3%. They found CFW has high sensitivity and thus is the best method to detect fungal agents from clinically suspected onychomycosis cases [23].

Dass et al. in their study on 150 patients with onychomycosis found that direct microscopy with KOH mount, calcofluor mount, and mycological culture showed positive results in 84 (56%), 95 (63.33%), and 59 (39.33%), respectively. Mycological culture was the least sensitive method, and CFW was the most sensitive method among the three methods [21].

Punjabi et al. in their study to evaluate the staining efficacy of CFW and acridine orange for the detection of Candida species found that there is a statistically significant association between CFW fluorescent microscopy and the swab culture technique. The sensitivity and specificity of CFW were 85% and 50%, respectively [27]. Jahanshahi et al. also found similar results in their study of *Candida* infection in cases of oral squamous cell carcinoma [28].

As CFW also stains other tissue elements like collagen, keratin, and elastin, it needs to be differentiated from threads, mucus strands, epithelial cells, and crystals. The type and intensity of fluorescence help to differentiate them. The advantages of CFW stain are that it is a simple and rapid technique. Light microscopy of a crush smear takes a minimum of four to five minutes for screening each slide, while under fluorescent light, calcofluor white stain preparation can be viewed or screened easily on a low power objective (10X), and it can be completed within one minute. Background or debris material can also be easily differentiated based on fluorescent properties. Calcofluor white is safe to use in the laboratory and does not have any health hazards. The disadvantage is that it is expensive due to the need for a fluorescent microscope. Also, every setup may not have the cryostat for the frozen section and fluorescent microscope in the same laboratory. The tissue sections need to be evaluated as soon as they are stained, as with time, the amount of fluorescence reduces [27].

The limitation of our study is the small sample size (as thankfully the second wave receded). However, the work is ongoing and being used for diagnosis in bronchoalveolar lavage (BAL) and sputum samples too.

## Conclusions

Calcofluor white stain is a time-saving, specific, and sensitive technique for rapid detection of rhinocerebral mucormycosis. It can be used as an adjunct for intraoperative diagnosis of fungal infections, but not as a primary method of diagnosis. This stain is versatile and can be used with tissue sections, direct smears, and other clinical specimens. When the fungus is detected by CFW stain, a rapid diagnosis can be given even in the absence of classical forms on frozen sections. Rhinocerebral mucormycosis is a potentially life-threatening disease with severe morbidity. Hence, the use of all available modalities for rapid and early diagnosis is recommended.
